# Spatial Intuition in Elementary Arithmetic: A Neurocomputational Account

**DOI:** 10.1371/journal.pone.0031180

**Published:** 2012-02-13

**Authors:** Qi Chen, Tom Verguts

**Affiliations:** 1 Moss Rehabilitation Research Institute, Albert Einstein Healthcare Network, Philadelphia, Pennsylvania, United States of America; 2 Department of Experimental Psychology, Ghent University, Ghent, Belgium; King Abdullah University of Science and Technology, Saudi Arabia

## Abstract

Elementary arithmetic (e.g., addition, subtraction) in humans has been shown to exhibit spatial properties. Its exact nature has remained elusive, however. To address this issue, we combine two earlier models for parietal cortex: A model we recently proposed on number-space interactions and a modeling framework of parietal cortex that implements radial basis functions for performing spatial transformations. Together, they provide us with a framework in which elementary arithmetic is based on evolutionarily more basic spatial transformations, thus providing the first implemented instance of Dehaene and Cohen's recycling hypothesis.

## Introduction

Both professional mathematicians and the broader population think about numbers in spatial terms (e.g., [1]). Accordingly, many studies have suggested that there is a link between number and space [2]. This link is conveniently summarized by saying that numbers are internally represented on a spatially oriented “mental number line”. A recent finding suggested that this is more than a metaphor [3]. McCrink and colleagues asked participants to perform nonverbal elementary arithmetic operations (addition and subtraction). The participants were able to do this [4]. Importantly, their responses also showed an *operational momentum* (OM) effect, meaning that answers to addition problems were systematically overestimated and answers to subtraction problems were systematically underestimated. The interpretation favored by most authors [3], [5] is that numbers are shifted too far to the right on the mental number line with addition, and shifted too far to the left with subtraction. Because the OM effect seems to reinforce the idea of a spatial mental number line, it is currently attracting a lot of attention (e.g., [6], [7], [8]). However, it is important to distinguish this explanation from the effect itself. For this reason, we will refer to the OM effect when we talk about the empirical data (without interpretation); when we write operational momentum, this will refer to this interpretation. One problem with the operational momentum account is that it predicts no difference between symbolic and nonsymbolic numbers. However, Knops et al. [5] observed that the OM effect is larger for nonsymbolic numbers than for symbolic numbers. Further, it remains unclear how the shifting operation on the mental number line is performed.

McCrink et al. [3] also shortly noted an alternative explanation for the OM effect. In particular, operations could be “accidentally” performed on a compressed logarithmic scale, and are therefore over- and underestimated for addition and subtraction, respectively. For example, if instead of adding numbers n1 and n2, subjects add log(n1) and log(n2), the result will be log(n1)+log(n2) = log(n1*n2) and so after the logarithm is undone, the result will be n1*n2 (which is usually an overestimation of n1+n2). This account suffers from the technical problem that, for addition, it predicts underestimation when n1 or n2 equals 1. This can be remedied, however, by assuming a less strongly compressed scale (e.g., power compression). Another problem with this account is that it does not specify how the brain implements addition and subtraction operations. Despite these issues, we believe that the core of this explanation, namely, a “noncompressed” operation (e.g., addition) in conjunction with a compressed representation, provides a fruitful way of thinking about the OM effect, and this idea will be one important part in the model to be developed.

Related to the OM effect is the space-operation association of responses (SOAR) effect, a term we use to encompass a behavioral and a neural observation [5], [6]. The behavioral effect is obtained in a paradigm where subjects see two successive numbers (presented as sets of dots (nonsymbolic number) or Arabic numerals (symbolic number)), mentally calculate their approximate sum or difference, and afterwards choose the closest number from a number of options. It is found that subjects prefer selecting options at a right location for addition tasks and at a left location in subtraction. At the neural level, Knops et al. [6] observed in a functional magnetic resonance imaging (fMRI) study that the activation pattern in parietal cortex during addition resembles the activation pattern produced by rightward eye movement, whereas the activation pattern during subtraction resembles the activation pattern corresponding to leftward eye movement. Together, the OM effect and the behavioral and neural aspects of the SOAR effect suggest an interaction between arithmetic and space. Unfortunately, a theoretical integration of these data, or of these data with related number-space interactions, is lacking. Providing one is the purpose of the current paper.

We combine two earlier modeling frameworks for parietal cortex. The first is a model we recently proposed on number-space interactions [9]. Here, number representations (in humans, residing in the horizontal part of the intraparietal sulcus (hIPS); [10]) become connected to spatial representations. The latter are proposed to reside in (the human homologue of) areas ventral intraparietal area (VIP) or lateral intraparietal area (LIP), areas coding for multimodal spatial representations [11]. A second model proposes that parietal cortex (e.g., LIP, VIP) implements radial basis functions for performing spatial transformations (e.g., vector addition; [12]). Different spatial representations are mapped onto radial basis function neurons, and transformation on the original spatial representations is implemented by projections from the radial basis function neurons to different spatial representations. This theory is supported by much neurophysiological evidence [13]. An example is the transformation from visual information in eye-centered coordinates to head-centered coordinates, which is useful for correctly turning the head toward a seen object. Another example, involving three collinear objects A, B, and C, is the estimation of the distance between objects A and C as the added distances between A and B and between B and C.

Here, we merge these two modeling frameworks to provide a neurocomputational account of elementary arithmetic. The resulting Spatial Arithmetic Model (SAM) deals with the OM and SOAR effects. We first set up a radial basis function network to implement spatial transformations [12]. Importantly, spatial transformations can often be performed by vector addition or vector subtraction: Calculation of the target destination after movement (cf. the previous paragraph) is an example. We argue that such spatial transformation networks are recycled for elementary arithmetic [14]. For this purpose, number representations are mapped onto the spatial representations that serve as input to the radial basis function network. In our earlier paper, we motivated such a mapping, and demonstrated that it allows accounting for many data on number-space interactions [9]. We use the same assumption here.

At this point, it is important to distinguish two different and independent types of compression. The first is the (possibly logarithmic) compression in number representation itself (e.g., [15]); this is not the focus of the current paper. The second, and relevant for the current paper, is the mapping between number representations and space. The mapping between nonsymbolic numbers and space is thought to be compressed [16], [17], so we implemented compression for the mapping between nonsymbolic numbers and space also. A power compression is easier to parametrize than log compression [18], so we opted for power compression for the mapping from nonsymbolic number to space. Interestingly, a recent report suggests that the mapping between number and space is well characterized by a power function [19]. However, it is important to note that the basic OM effect follows from the model implemented with either type of mapping (logarithmic or power). The mapping between symbolic numbers and space has been argued to be much less compressed than the mapping between nonsymbolic numbers and space (or even linearly spaced; [16], [17]), so we drastically reduced the power compression factor for the mapping between symbolic numbers and space.

## Methods

### Network architecture

**Figure 1:**
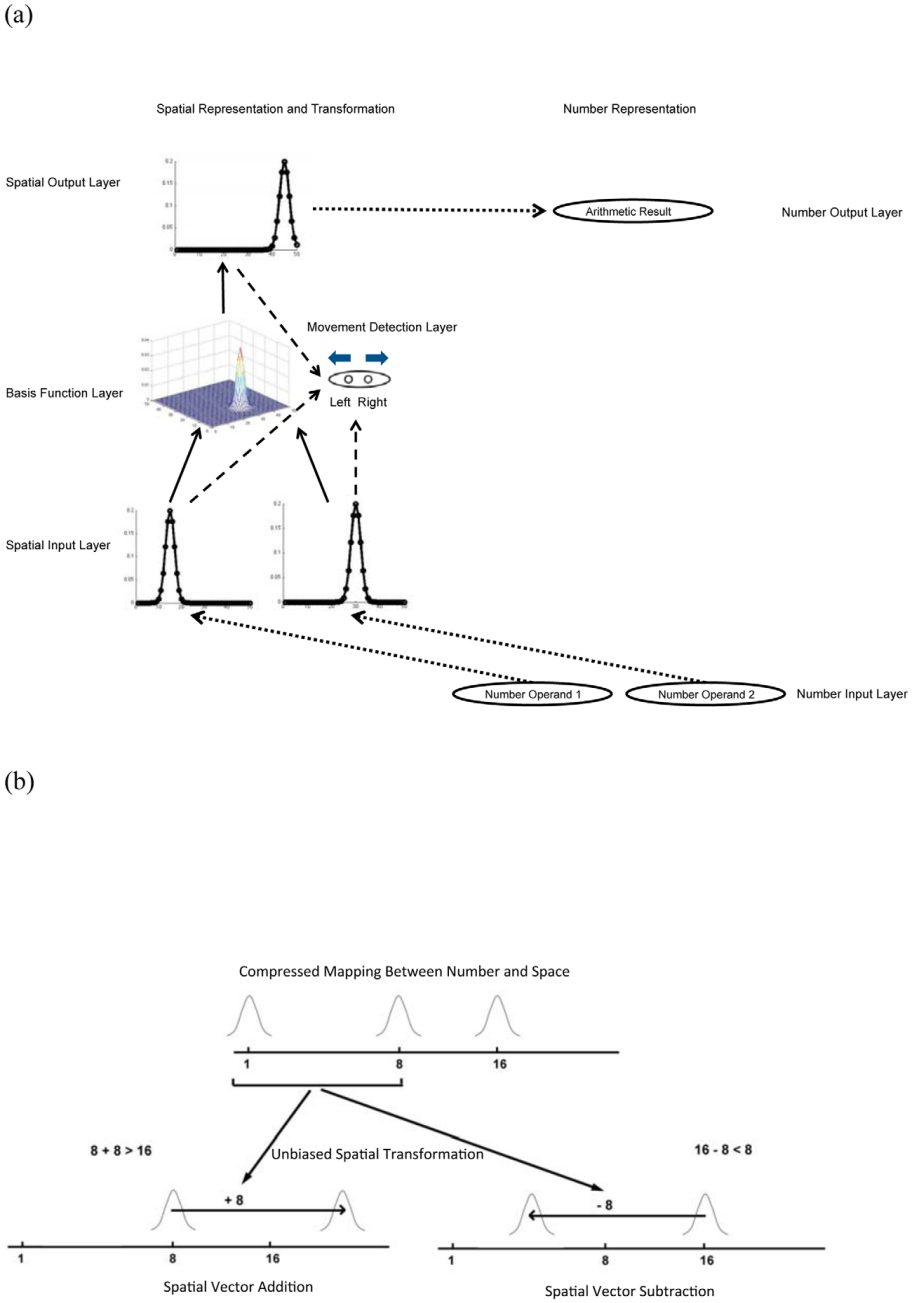
Schematic diagram and operation of the Spatial Arithmetic Model (SAM). (a). The two operands of an arithmetic problem are mapped onto two spatial input layers (dotted arrows) by a power compression function. The basis function layer combines these two inputs and sends activation to the spatial output layer. Then the activation from the spatial output layer is transformed to the corresponding arithmetic result (dotted arrow). In addition, spatial representations are sent to a movement detection layer (dashed arrows). (b). Addition is implemented by spatial vector addition on a compressed mapping between number and space, leading to overestimation; subtraction is implemented by spatial vector subtraction on a compressed mapping between number and space, leading to underestimation.

The SAM model architecture is shown in Figure 1a. Its core is a three-layer feedforward structure, consisting of 600 input, 90000 hidden, and 300 output neurons.

There are two groups of 300 input neurons. Presentation of an object to such an input layer results in a Gaussian activation curve. In particular, each input neuron is maximally activated by a preferred spatial location, *p*, and the activation value of each input neuron is based on the distance between this preferred spatial location and the actual object location, *s*, according to a Gaussian function (localist but smoothed representation; see Figure 1a):
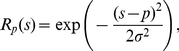
(1)where 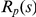 is the activation of the spatial input neuron with preferred position *p* when a target appears at position *s*.

Each neuron in the hidden layer receives input from a unique combination of one neuron from each of the two layers of input neurons, so the hidden layer comprises 90000 basis function neurons. The activation value of a hidden layer neuron equals the product of its input neurons' activation values:
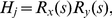
(2)where 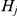 is the activation of the hidden unit receiving input from two input neurons from the two groups, with preferred location *x* and *y*, respectively.

The output layer of 300 neurons produces another spatial representation. Every output neuron receives inputs from all hidden neurons as follows:
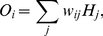
(3)where 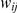 represents the synaptic connection weight from hidden neuron *j* to output neuron *i*.

Finally, we added a movement detection layer containing two neurons (left and right) to simulate movement detection. For performing spatial arithmetic, number input and output representations were attached to this core spatial model (dotted arrows in Figure 1a).

### Simulation

For simplicity, we implemented addition and subtraction with the same network architecture (Figure 1a), but with a different output layer (and connection weights) for the two operations. However, it is possible to integrate them with a single output layer either by using recurrent connections [12] or by gain modulation provided by task demand units [20].

The weights between the hidden layer and the output layer are chosen such that they minimize the average squared error between intended and actual spatial responses. We obtained these weights by solving the matrix weight equations for the original spatial transformations. This entails the assumption that the mapping from retina to space is noncompressed, or at least less compressed than the mapping from number to space. The model's performance is perfect using these optimal weights, so there is no momentum effect for spatial transformations.

The weights from the spatial layers to the movement detection neurons (dashed arrows in Figure 1a) were set accordingly. Specifically, linearly monotonically increasing weights were chosen from each spatial input layer (the original spatial position of a moving target) to the “left movement” neuron. In this way, a more leftward object at input induces a weaker response in the “left movement” motor neuron. In contrast, linearly monotonically decreasing weights were chosen from the spatial output layer to the “left movement” neuron; hence, a more leftward positioned object at output induced a stronger “left movement” response. The reverse pattern of weights was chosen for connections to the “right movement” neuron. As a result, if an object at input is more leftward than the same object at output, a “right movement” response is elicited. The activation of the movement detection neurons can then be used for making appropriate eye movements. Other (than linear) monotonic weight patterns lead to similar results as those reported here. On each trial, the output layer and one randomly chosen input layer passed activation to the movement detection layer.

For the mapping between nonsymbolic numbers and space, the (power) compression had an exponent of 0.8. For the mapping between symbolic numbers and space (which should be less compressed, as argued above), the exponent was 0.95. The qualitative pattern of results does not depend on these exact values. After numbers were transformed from number input to the spatial input layers, the information passed through the radial basis function network, and the activation pattern from the spatial output layer was read out. Then, the spatial output information was transferred to the corresponding number representation. In a sense, then, the linear transformation network “ignores” the number-to-space compression, which generates the OM effect.

For the behavioral effects, we focus on Experiment 2 of Knops et al. [5]. This study provides the most direct evidence for the OM effect because the results of the operations were constant for addition and subtraction. As in that study, we chose a set of numbers (19, 21, 25, 35, 41, and 49) as results for both addition and subtraction. The corresponding operands are the same as in the original data (Table 2 of Knops et al., [5]). Similar results are obtained if the operands rather than the results remain constant (Knops et al., Experiment 1).

## Results

### The OM effect

**Figure 2:**
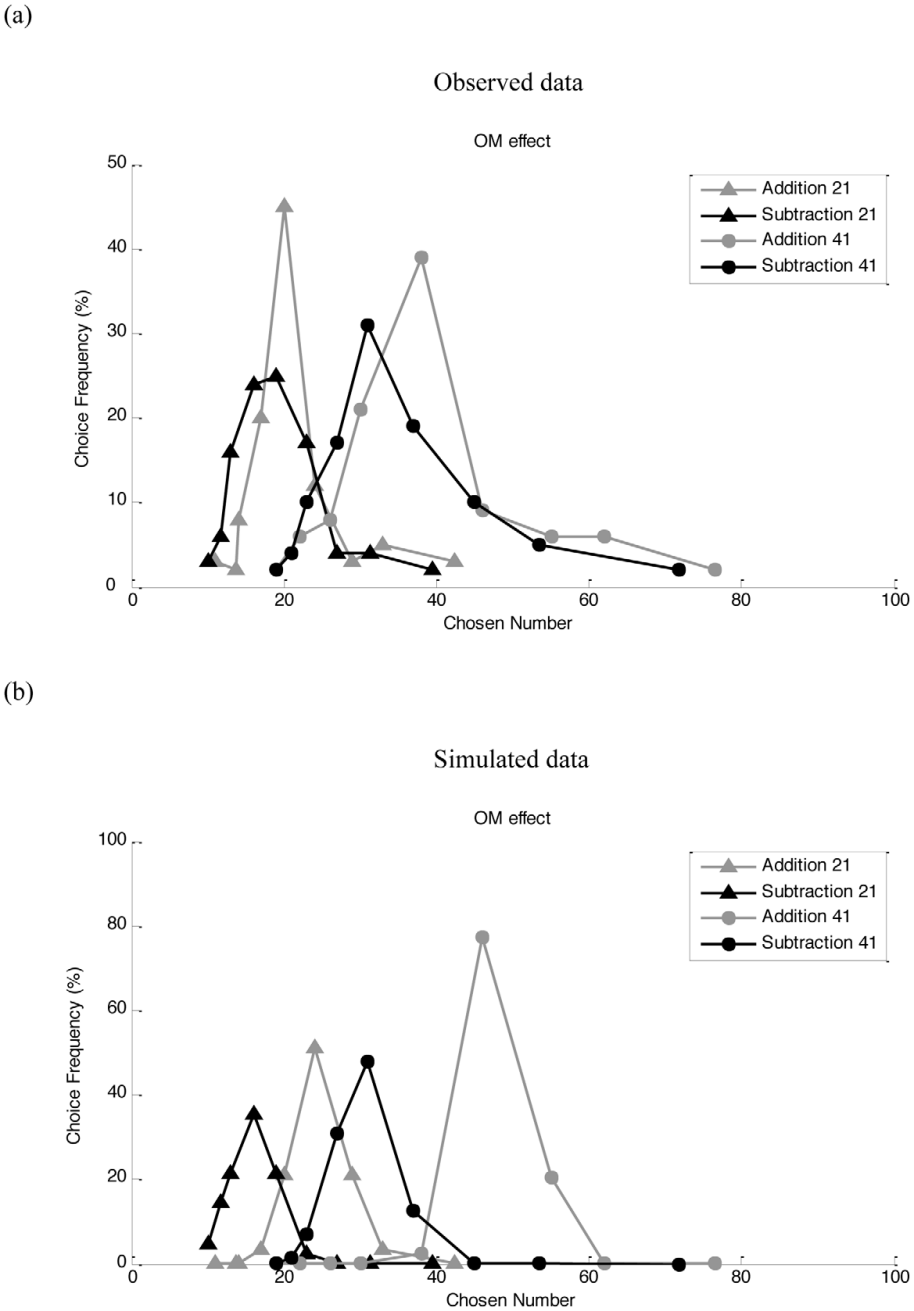
The OM effect. Observed data (2a, from [**5**]) and simulated data (2b). Observed data represents the distribution of the responses to two nonsymbolic addition and subtraction problems (with arithmetic results of 21 or 41). For the simulated data, choice frequency of a number is proportional to the activation of that number. In each case, the OM effect is reflected by a leftward bias in subtraction, compared with addition problems.

**Figure 3:**
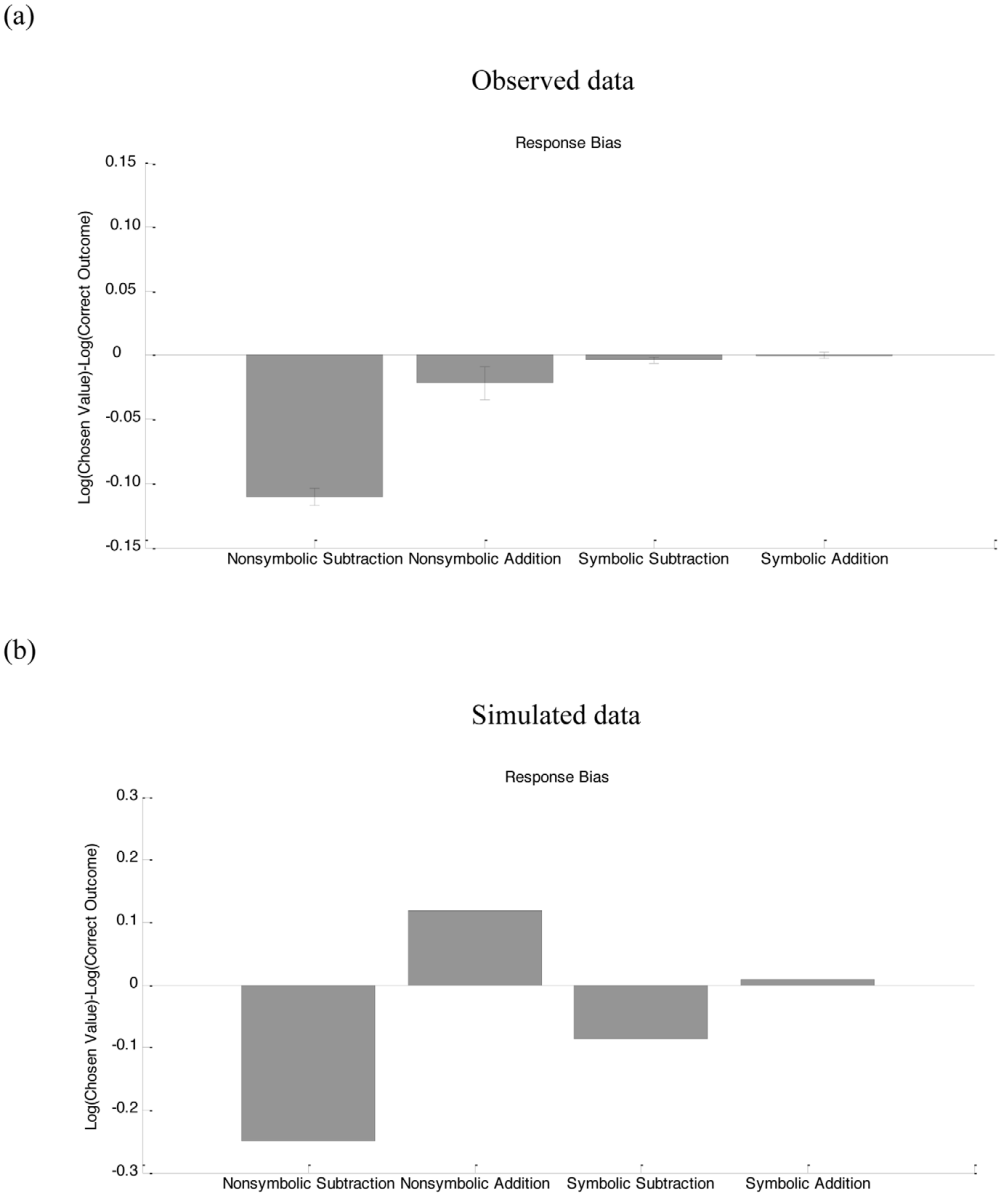
The mean response bias for nonsymbolic and symbolic arithmetic problems. Observed data (3a, from [5]) and simulated data (3b). This bias reflects the average difference between the chosen result and the correct value, both represented on a log scale (following [5]). A negative bias indicates underestimation, and a positive bias indicates overestimation.

For nonsymbolic number, representative simulation results are shown in Figure 2b (empirical data in Figure 2a). These curves represent the model's choice frequencies in response to different nonsymbolic addition and subtraction problems. The model tends to select larger numbers for addition than for subtraction, even when the results of addition and subtraction problems are the same (i.e., the OM effect). Bias sizes for all simulated numbers are shown in Figure 3b (empirical data in Figure 3a). There is a bias toward smaller numbers for subtraction and a bias toward larger numbers for addition. Importantly, when training the radial basis networks, there was no compression. The OM effect emerges from the combination of a compressed number-to-space mapping with an unbiased transformation in the radial basis network (Figure 1b).

Simulated data for symbolic subtraction and addition is shown in Figure 3b. The model tends to show an OM effect, but, in line with empirical data ([5]; Figure 3a) the bias is significantly reduced relative to nonsymbolic numbers. One difference with the empirical data is that we obtained no bias toward small numbers (Figure 3a). However, as noted by Knops et al., there can simply be an overall bias toward responding with smaller numbers which was not modeled here.

### The SOAR effect

**Figure 4:**
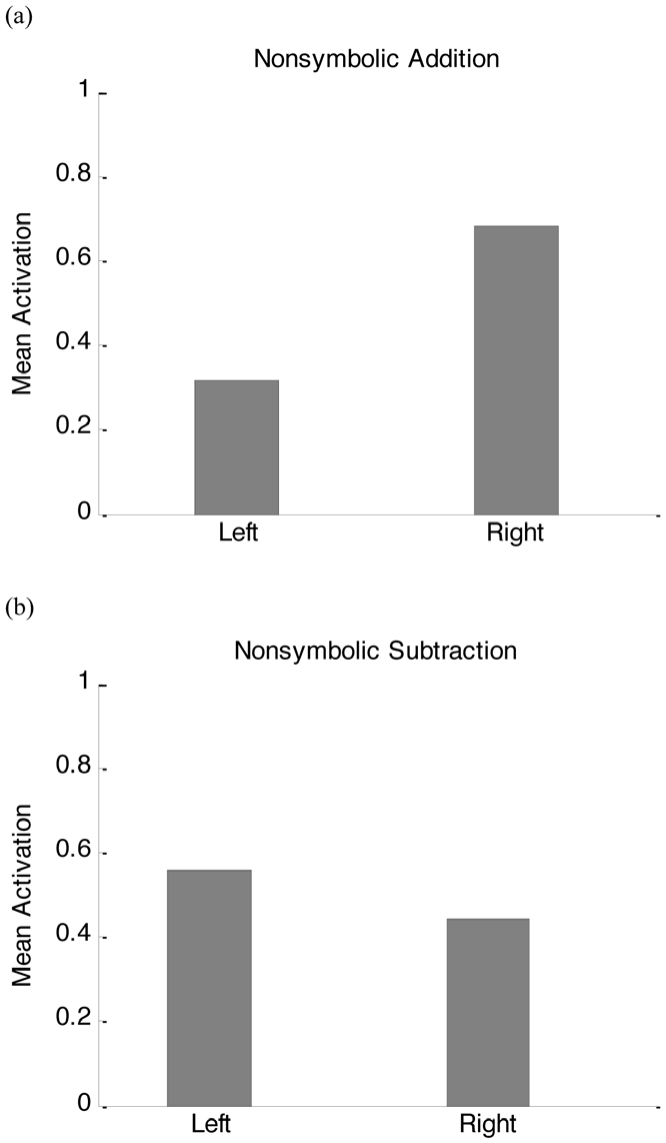
The SOAR effect. Mean activation of the left and right neurons in the movement detection layer was calculated across addition and subtraction problems separately. 4a: nonsymbolic addition; 4b: nonsymbolic subtraction.

The spatial preference for nonsymbolic addition and subtraction is shown in Figure 4. The model tends to activate the right movement neuron for addition (Figure 4a) and the left movement neuron for subtraction (Figure 4b). To model the behavioral and neural aspect of the SOAR effect explicitly, an appropriate decision and response mechanism should be introduced, linking the movement neurons to spatial attention and eye movement representations further downstream. In this way, the movement neuron activation can explain the behavioral aspect of the SOAR effect in the sense that a shift of attention to the left side of space would favor the left side in the competition for a decision [21], thus leading to more leftward choices. It can also explain the neural aspect of the SOAR effect [6] in that a pattern recognition device that would be trained to distinguish left from right eye movements would instantaneously (i.e., without extra training) generalize to arithmetic operations as subtraction versus addition, respectively. This is exactly what Knops et al. obtained. Furthermore, Knops et al. observed that classification performance is better for addition than for subtraction. This asymmetry can also be explained by our model as left and right are much more differentiated in addition than in subtraction (absolute difference in activation is 0.36 for addition but only 0.12 for subtraction; see Figure 4). The reason for this is that movement is consistently to the right for addition (e.g., 4+3 = 7); in contrast, movement is not consistently leftward, but can be to the right also for subtraction, depending on which operands are chosen (e.g., in 7−3 = 4, movement from 3 to 4 is “to the right”).

## Discussion

We proposed a model to account for the striking similarities between arithmetic and space. Building on our earlier number-space model [9], we implemented the hypothesis that the OM and SOAR effects emerge from a mapping between number and space. Furthermore, basis function networks for spatial transformations are recruited for elementary arithmetic by mapping numbers onto the input layers of the basis functions. The basis function layer, corresponding to multimodal parietal areas such as LIP or VIP, plays a key role for numerical arithmetic also. Because these areas are used for saccadic and attentional control, the networks involved in shifting attention are recycled for elementary arithmetic. This constitutes an instance of Dehaene and Cohen's [14] recycling hypothesis and, more broadly, of the embodied cognition hypothesis [22].

Our model is not neurobiologically plausible in the sense that an explicit error signal is injected to learn the mapping task. However, the pattern of optimal weights for generating the correct spatial responses can emerge spontaneously through random spatial movements and correlation-based synaptic modification [23]. In this sense, the injection of error should not be problematic. Another possible departure from neurobiology is the fact that the hidden layer was much bigger than the input layers. However, this simplification is also well-motivated. First, the input layer only represents an approximation to the actual input system. Second, the hidden layer would probably also approximate the required input-output function with a less extensive set of basis functions. However, because this issue is beyond the scope of the current paper, we simply implemented the complete set of basis functions.

On a more conceptual level, it is important to explain why learning during the task does not abolish the bias and hence the OM effect. One of advantages of the basis function network is that it learns the correct transformation efficiently and correctly [20]. Hence, it is easy to learn the correct transformation for the compressed representation without any bias (OM effect). However, under the recycling hypothesis [14], basic cognitive processes are recruited for high-level cognition. When applied to the current context, this implies that a network used for space processing is recruited for number processing, a much less frequent task. Accordingly, we first set up a radial basis function network to implement spatial transformation, not because a separate numerical transformation network would be hard to learn for the organism, but simply because nonsymbolic number addition or subtraction is so infrequent. In other words, it seems unlikely that subjects would set up a new network just for the current task. However, the model does predict that, with frequent practice in the nonsymbolic number task, and when subjects improve due to feedback, effects on spatial processing should be discernible. This remains a prediction for future experimental investigation.

We propose that radial basis function networks trained for one purpose (spatial transformations) are recycled for other tasks (elementary arithmetic) when these other tasks are less frequent. Such basis function networks are found throughout parietal cortex for performing tasks such as reaching and grasping [24]. This may be why numbers “reside” in these same parietal circuits. However, when a task is extensively trained, it probably receives its own dedicated structures (radial basis function networks). One example is symbolic multiplication (see [25], for a basis function network of mental multiplication). Accordingly, the model may not only provide an explanation of the OM and SOAR effects, but more generally a perspective on the interaction between cognitive and more basic operations in parietal cortex.

## References

[1] Galton F (1880). Visualised numerals.. Nature.

[2] Hubbard EM, Piazza M, Pinel P, Dehaene S (2005). Interactions between number and space in parietal cortex.. Nature Reviews Neuroscience.

[3] McCrink K, Dehaene S, Dehaene-Lambertz G (2007). Moving along the number line: Operational momentum in nonsymbolic arithmetic.. Perception & Psychophysics.

[4] Cordes S, Gallistel CR, Gelman R, Latham P (2007). Nonverbal arithmetic in humans: Light from noise.. Perception & Psychophysics.

[5] Knops A, Viarouge A, Dehaene S (2009). Dynamic representations underlying symbolic and nonsymbolic calculation: Evidence from the operational momentum effect.. Attention, Perception, & Psychophysics.

[6] Knops A, Thirion B, Hubbard EM, Michel V, Dehaene S (2009). Recruitment of an area involved in eye movements during mental arithmetic.. Science.

[7] Pinhas M, Fisher MH (2008). Mental movements without magnitude? A study of spatial biases in symbolic arithmetic.. Cognition.

[8] McCrink K, Wynn K (2009). Operational momentum in large-number addition and subtraction by 9-month-old infants.. Journal of Experimental Child Psychology.

[9] Chen Q, Verguts T (2010). Beyond the mental number line: A neural network model of number-space interactions.. Cognitive Psychology.

[10] Dehaene S, Spelke E, Pinel P, Stanescu R, Pinel P (1999). Sources of mathematical thinking: Behavioral and brain-imaging evidence.. Science.

[11] Anderson R, Cui H (2009). Intention, action planning, and decision making in parietal-frontal circuits.. Neuron.

[12] Deneve S, Latham PE, Pouget A (2001). Efficient computation and cue integration with noisy population codes.. Nature neuroscience.

[13] Pouget A, Snyder LH (2000). Computational approaches to sensorimotor transformations.. Nature Neuroscience.

[14] Dehaene S, Cohen L (2007). Cultural recycling of cortical maps.. Neuron.

[15] Dehaene S, Haggard P, Rossetti Y, Kawato M (2007). Symbols and quantities in parietal cortex: Elements of a mathematical theory of number representation and manipulation.. Attention and Performance XXII. Sensori-motor foundations of higher cognition.

[16] Dehaene S, Izard V, Spelke E, Pica P (2008). Log or linear? Distinct intuitions of the number scale in Western and Amazonian indigene cultures.. Science.

[17] Siegler RS, Opfer JE (2003). The development of numerical estimation: Evidence for multiple representations of numerical quantity.. Psychological Science.

[18] Viarouge A, Hubbard EM, Dehaene S, Sackur J (2010). Number line compression and the illusory perception of random number.. Experimental Psychology.

[19] Barth H, Paladino AM (2011). The developmental of numerical estimation: Evidence against a representational shift.. Developmental Science.

[20] Salinas E (2004). Fast remapping of sensory stimuli onto motor actions on the basis of contextual modulation.. Journal of Neuroscience.

[21] Desimone R, Duncan J (1995). Neural mechanisms of selective visual attention.. Annual Review Neuroscience.

[22] Barsalou LW (1999). Perceptual symbol systems.. Behavioral and Brain Sciences.

[23] Salinas E, Abbott LF (1995). Transfer of coded information from sensory to motor networks.. Journal of Neuroscience.

[24] Gottlieb J (2007). From thought to action: The parietal cortex as a bridge between perception, action, and cognition.. Neuron.

[25] Verguts T, Fias W (2005). Interacting neighbors: A connectionist model of retrieval in single-digit multiplication.. Memory & Cognition.

